# Circadian motor activity of non-dominant hand reaches acrophase later than dominant hand

**DOI:** 10.1038/s41598-022-09717-5

**Published:** 2022-04-06

**Authors:** Vincenzo Natale, Marco Fabbri, Monica Martoni, Lorenzo Tonetti

**Affiliations:** 1 Department of Psychology “Renzo Canestrari”, University of Bologna, Viale Carlo Berti Pichat 5, 40127 Bologna, Italy; 2 Department of Psychology, University of Campania Luigi Vanvitelli, Caserta, Italy; 3 Department of Experimental, Diagnostic and Specialty Medicine, University of Bologna, Bologna, Italy

**Keywords:** Physiology, Psychology, Neurology

## Abstract

Motor activity during the first half of nocturnal sleep is lateralized to the non-dominant hand. What remains is to determine which account could explain this phenomenon: the more pronounced homeostatic deactivation of the dominant hemisphere or the circadian asymmetry in the hemispheric activation. To better understand the nature of these motor asymmetries, we performed an ecological study assessing the circadian motor activity in 34 evening, 52 intermediate, and 27 morning types. We observed a significant circadian phase delay of the 24-h motor activity pattern of the left hand in comparison to the right hand, regardless of chronotype. Moreover, we replicated higher motor activity in the left hand in comparison to the right hand in late evening that reached statistical significance only in evening and intermediate types. Analysing motor activity around bedtime and wake-up time, we observed a reverse pattern between circadian typologies: evening types showed higher activity in the left hand in comparison to the right hand before bedtime, while morning types showed significantly higher motor activity in the right hand in comparison to the left after wake-up time. Results support the hypothesis of a different circadian phase relationship between the two hemispheres.

## Introduction

Since the seventies, it has been documented that during the first half of nocturnal sleep the non-dominant hand is more active in comparison to the dominant one^1^. This phenomenon has been consistently replicated and appears relatively independent of the NREM/REM (Non-Rapid Eye Movement/Rapid Eye Movement) sleep cycle^2–4^. However, to date there are no shared explanations for such a phenomenon. We currently know that this particular motor asymmetry can be also documented before sleep onset^5,6^, suggesting that sleep is not a condition *sine qua non*.

According to the two-process model of sleep regulation, biological and behavioural circadian variations are driven by two interacting processes: the homeostatic process (S), which increases sleep debt with time spent awake and the circadian process (C), which drives a near 24-h endogenous rhythm^7^.

Within this theoretical framework, referring to the S process, it has been suggested that the relative superiority of the non-dominant hand during sleep could derive from a more pronounced homeostatic deactivation of the dominant hemisphere^8^. In other words, being more active during wakefulness, the dominant hemisphere could present a greater increase in the sleep debt compared to the non-dominant hemisphere and, therefore, would fall asleep more deeply than the non-dominant one. This hypothesis has been tested with the use-dependent recovery function paradigm. Kattler, Dijk and Borbély^9^ induced an additional passive movement by vibratory stimuli to the hand for six hours before bedtime and found greater Slow Wave Activity (SWA—a physiological marker of sleep homeostasis^10^) in the contralateral hemisphere during the first hours of sleep in comparison to the baseline. In the same way, Huber and collaborators^11^ were able to show that arm immobilization during wakefulness for twelve hours caused a local decrease in SWA in subsequent sleep episodes. In a prospective study, actigraphic data were collected before, during, and after a night without sleep^12^. However, the greater motor activity of the non-dominant hand late in the evening did not increase after sleep deprivation, as predicted by the use-dependent recovery function model, but, in fact, disappeared. Circadian motor activity of the right and left hand was also studied in a sample of right- and left-handed participants^13^. According to the use-dependent recovery function model, a reversed pattern of motor activity asymmetries between right- and left-handed participants was supposed, however, once again, the results did not support this.

To explain the shift in dominant and non-dominant hand motor activity in the early hours of nocturnal sleep, an alternative hypothesis focussing on the C process has been advanced: the shift could derive from a different circadian phase relationship between the two hemispheres^6^. This hypothesis was backed up by results obtained analysing diurnal performance oscillation: cognitive performances in verbal tasks (involving predominantly the left hemisphere) are better during the morning, while cognitive performances in spatial tasks (involving predominantly the right hemisphere) are better in the evening^14,15^. Such a hypothesis agrees also with an underrated result of the experiment conducted by Kattler and co-authors^9^: after left-hand somatosensory stimulation, the interhemispheric asymmetry index during the first sleep cycle still shifted to the left hemisphere. Moreover, although both right- and left-hemisphere light stimulations attenuated subjective alertness, only the stimulation of the right visual cortex was able to trigger a significant reduction in EEG (electroencephalogram) delta activity^16^. Much research^6,12,13^ has shown that the mean motor activity in the left hand is higher in comparison to the right between 10:00 p.m. and 11:00 p.m., as if there was some sort of a key time window. Moreover, in right-handed participants, the right hand reached its acrophase slightly but significantly earlier than the left hand (around 12 min)^6^. Indeed, such data agree with the hypothesis of the existence of a dual circadian pacemaker in the nervous system, which has been proposed several times over the years based on results deriving from biological experiments performed both in animals^17^ and humans^18^.

To separate the role of S and C processes, different experimental paradigms, such as forced desynchrony or constant routine, are possible^19^. However, these paradigms pose certain problems in evaluating behavioural variables. For this reason, alternative ecological paradigms have been put forward, such as a chronotype-based paradigm that considers the chronotype as an independent variable^20^. It has also been suggested that chronotype could constitute a unique tool to access the interplay between the S and C processes under normally entrained day-night conditions^21^. To further investigate ecologically the role of S and C processes on circadian motor activity asymmetry, we decided to analyse the circadian motor activity of evening, intermediate, and morning types. It is well known that sleep quantity and sleep quality do not distinguish chronotypes, while they differ in the phase of sleep^22^: morning types go to bed earlier then intermediate types, and intermediate types go to bed earlier than evening types, while for the wake-up time, the reverse pattern is recognised. Therefore, if circadian motor asymmetries are prevalently driven by a S process, we may expect a similar pattern between chronotypes in the late evening, i.e., a higher level of motor activity in the non-dominant hand in comparison to the dominant hand, regardless of bedtime. By contrast, if circadian motor asymmetries are prevalently driven by a C process, we may expect a different pattern between chronotypes, regardless of sleep condition. In other words, motor asymmetries would always be present around 10 p.m., but they would be less evident in morning types because around that time they are already asleep (masking effect). Conversely, motor asymmetries late in the evening should be more evident in evening types because they usually go to bed later than 10 p.m. In order to better understand the role of sleep condition, we examined motor activity near the phases of wake/sleep and sleep/wake transition, regardless of the specific time of day they occurred. Once again, if circadian motor asymmetries are prevalently driven by the S process, we may expect a similar motor asymmetry late in the evening between the chronotypes. By contrast, if circadian motor asymmetries are prevalently driven by the C process, we may expect a different pattern between the chronotypes with a clearer higher mean motor activity in the left hand in evening types.

## Methods

### Participants

A sample of 113 healthy university students (41 males, 72 females), mean age = 23.9 ± 4.2, were enrolled as volunteers in the study. After a brief description of the study, participants read and signed a written informed consent form. All participants were all right-handed, as assessed by the Italian version^23^ of the Edinburgh Handedness Inventory^24^. Participants were classified as evening, intermediate, or morning types according to the cut-off scores of the Italian version^25^ of the reduced Morningness-Eveningness Questionnaire (MEQr)^26^, i.e., 4–10, 11–18, and 19–25, respectively. The MEQr is composed of 5 items taken from the 19-item version of the MEQ^27^. Since MEQr proved to have good external validity and good discriminant ability between extreme chronotypes, its use has been proposed within the research field^28^. The MEQr was given at the end of the study so that participants’ behaviour would not be influenced in any way.

### Actigraphy and procedure

Actigraphic recordings were obtained using the Micro MotionloggerWatch actigraph (Ambulatory Monitoring Inc., Ardsley, NY). Devices were initialized for zero crossing mode to collect data in 1-min epochs. Participants wore an actigraph on each wrist for three consecutive nights (excluding Saturday and Sunday) to obtain at least 48 consecutive hours of reliable data. Participants were free to spend their daytime hours and sleep time out of the laboratory. They were also instructed to push the actigraph event marker to signal when they went to bed and woke up in the morning. Each participant provided the written informed consent before being enrolled in this study, carried out during the autumn 2021, that was approved by the Bioethics Committee of the University of Bologna (prot. n. 284786) and carried out in accordance with the Declaration of Helsinki.

### Actigraphic sleep parameters

To evaluate sleep features, actigraphic data were analysed using Action W-2 (version 2.7) software (Ambulatory Monitoring, Inc., Ardsley, NY). This software identified each epoch as sleep or wakefulness using the mathematical model validated by Cole and co-authors^29^. According to such model, sleep onset was defined as the first epoch of the first block of 20 min of persistent sleep, while sleep offset as the end of the last sleep episode within the interval of the time spent in bed. In order to examine the actigraphic sleep profile, we considered the following measures: the time the participants went to bed and switched off the light (bedtime) and the time the participants last woke up in the morning (wake-up time); total sleep time (TST) (sum, in minutes, of all sleep epochs between sleep onset and sleep end); sleep efficiency percentage (SE%) (the ratio of total sleep time to time in bed multiplied by 100). For each participant, the mean values were calculated over the three nights.

### Actigraphic motor activity

To evaluate motor activity, actigraphic data were extracted using the version 1.16 of Action 4 software (Ambulatory Monitoring, Inc., Ardsley, NY). The actigraphic recording was divided into 60-min intervals starting from 16:00 h and the hourly mean activity levels over the 24 h were calculated for each participant. Moreover, the MESOR and acrophase were computed using cosinor analyses, which are implemented within the Action 4 software. Cosinor analysis is a statistical technique specifically developed for the study of cyclic functions^30^. For each participant, the mean values were computed based on 48-h recordings.

We also extracted hourly motor activity data considering the hours close to bedtime (i.e., four hours before and four hours after bedtime) and wake-up time (i.e., four hours before and four hours after wake-up time).

### Statistical analyses

Gender and age differences in chronotype were explored with a chi-squared test and an analysis of variance, respectively.

The chronotype differences in actigraphic sleep parameters were analysed through a set of analysis of variance with chronotype as independent variable and each actigraphic sleep parameter as dependent variable.

To analyse the motor activity pattern during the 24 h, a mixed three-way analysis of variance (ANOVA) was performed: hand (two levels: right and left) (within-subjects factor); time of day (24 levels) (within-subjects factor); chronotype (three levels: morning, intermediate, evening type) (between-subjects factor).

As regards the acrophase and MESOR, we performed a mixed two-way ANOVA: hand (two levels: right and left) (within-subjects factor); chronotype (three levels: morning, intermediate, evening types) (between-subjects factor).

To analyse the motor activity pattern during the wake-sleep transition (bedtime), a mixed three-way ANOVA was performed: hand (two levels: right and left) (within-subjects factor); time (8 levels: four hours before and four hours after bedtime) (within-subjects factor); chronotype (three levels: morning, intermediate, evening types) (between-subjects factor).

To analyse the motor activity pattern during the sleep–wake transition (wake up time), a mixed three-way ANOVA was performed: hand (two levels: right and left) (within-subjects factor); time (8 levels: four hours before and four hours after wake-up time) (within-subjects factor); chronotype (three levels: morning, intermediate, evening types) (between-subjects factor).

## Results

Participants were assigned to one of the three groups: morning types (n = 27, 7 males and 20 females, 23.89%), intermediate types (n = 52, 20 males and 32 females, 46.02%) and evening types (n = 34, 14 males and 20 females, 30.09%). Setting the significance level to p < 0.05, the frequency distribution of circadian typology between males and females was not significantly different (χ^2^_2_ = 1.71, p = 0.42). Moreover, chronotype did not differ by age (F_2,110_ = 1.56—p = 0.21).

As expected, morning-types (00:11 a.m. ± 0:56) go to bed significantly earlier than intermediate types (01:04 a.m. ± 1:20), and the latter significantly earlier than evening types (01:49 a.m. ± 1:02) (F_2,110_ = 14.62—p = 0.00001). Likewise, morning types (08:01 a.m. ± 1:03) wake up in the morning significantly earlier than intermediate types (08:48 a.m. ± 1:18), and the latter significantly earlier than evening types (10:04 a.m. ± 1:31) (F_2,110_ = 15.44—p = 0.00001). Actigraphic sleep quantity (TST) and sleep quality (SE%) did not significantly differ by chronotype (respectively: 457 ± 55 min. morning, 432 ± 63 min. intermediate, 450 ± 57 min. evening types, F_2,110_ = 1.74—p = 0.18; 95.0 ± 6.0% morning, 95.5 ± 4.4% intermediate, 95.3 ± 5.3% evening types, F_2,110_ = 0.08—p = 0.92).

**Figure 1:**
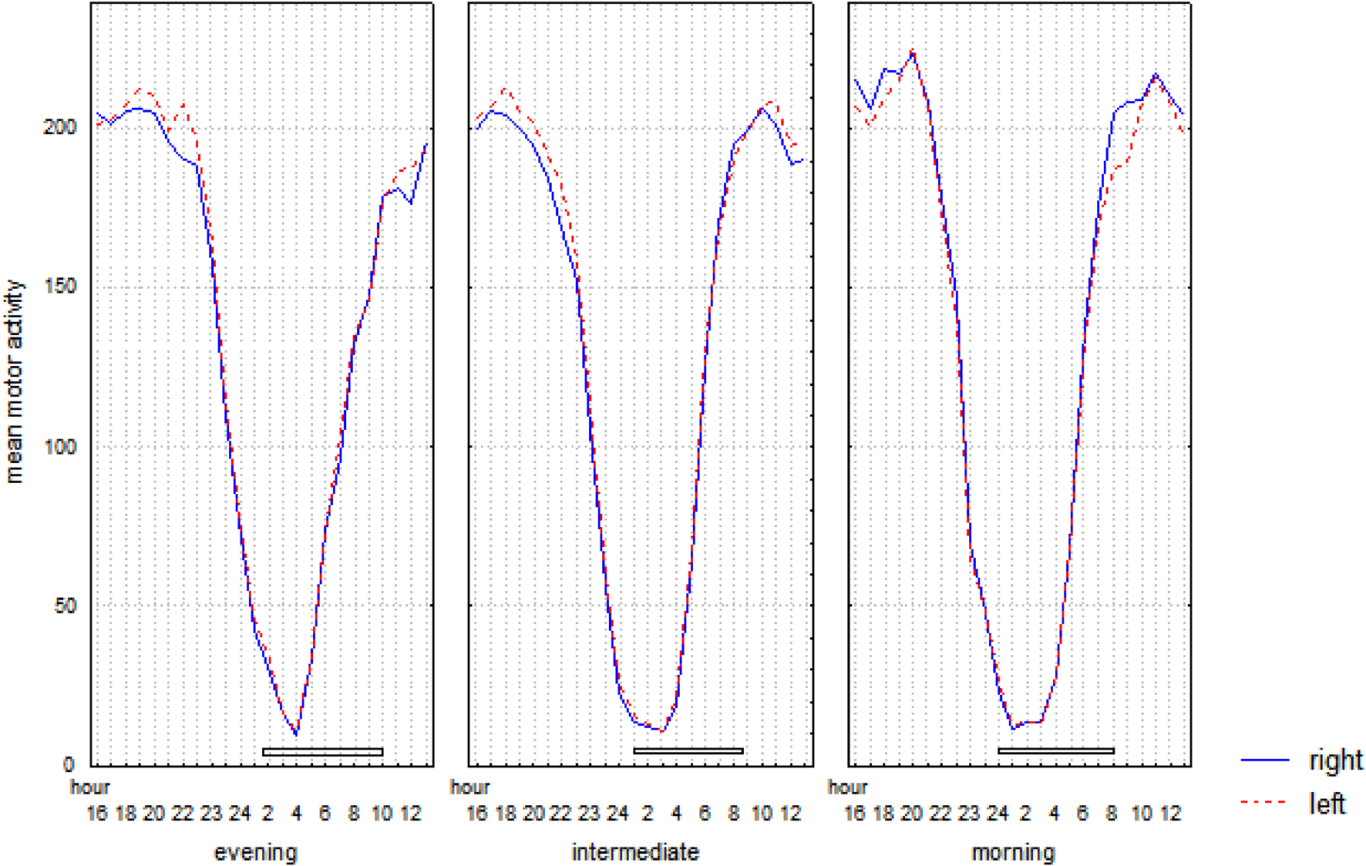
Hourly mean motor activity over the 24 h of the right and left hand in evening, intermediate, and morning types. The white horizontal bar represents the time spent in bed by each chronotype, with the extremes pointing to the bedtime and get-up time.

Results of the analyses on the motor activity pattern during the 24 h are summarized in Table 1a and shown in Fig. 1. Overall, results confirm a small but significant shift over 24 h. In particular, the mean motor activity of the non-dominant hand is higher at around 10:00 p.m. However, such an effect reaches statistical significance only in evening (t_33_ = − 2.33—p = 0.03) and intermediate types (t_52_ = − 3.27—p = 0.002). On the contrary, morning types show a significantly higher mean motor activity in the dominant hand at 10 a.m. (t_26_ = 2.33—p = 0.03) and 11 a.m. (t_26_ = − 2.49—p = 0.02).

**Table 1:** Statistics of the mixed three-way analysis of variance on motor activity pattern during the 24 h (circadian, a), wake-sleep transition (sleep onset, b), and sleep–wake transition (wake-up, c).

	F value	Significance
**(a) Circadian**		
1 chronotype	F_2,110_ = 0.51	p = 0.60
2 right-left hand	F_1,110_ = 0.34	p = 0.56
3 time of day	F_23,2530_ = 227.84	p = 0.00001
1 × 2	F_2,110_ = 1.57	p = 0.21
1 × 3	F_46,2530_ = 6.83	p = 0.00001
2 × 3	F_23,2530_ = 3.01	p = 0.00001
1 × 2 × 3	F_46,2530_ = 1.34	p = 0.06
**(b) Sleep onset**		
1 chronotype	F_2,110_ = 2.8	p = 0.06
2 right-left hand	F_1,110_ = 7.0	p = 0.009
3 time	F_7,770_ = 1349	p = 0.00001
1 × 2	F_2,110_ = 2.0	p = 0.13
1 × 3	F_14,770_ = 1.7	p = 0.06
2 × 3	F_7,770_ = 3.5	p = 0.001
1 × 2 × 3	F_14,770_ = 3.2	p = 0.0001
**(c) Wake up**		
1 chronotype	F_2,110_ = 0.3	p = 0.72
2 right-left hand	F_1,110_ = 0.9	p = 0.33
3 time	F_7,770_ = 1930.0	p = 0.00001
1 × 2	F_2,110_ = 2.4	p = 0.09
1 × 3	F_14,770_ = 0.9	p = 0.51
2 × 3	F_7,770_ = 4.6	p = 0.0001
1 × 2 × 3	F_14,770_ = 1.8	p = 0.03

As regards the acrophase, the first factor (hand) was significant (F_1,110_ = 129.22—p = 0.00001). In particular, the right hand reaches the acrophase significantly earlier (04:42 p.m. ± 0:49) than the left hand (05:02 p.m. ± 0:48). As expected, morning types reach their acrophase (04:01 p.m. ± 2:20) significantly earlier than intermediate types (04:44 p.m. ± 1:24), and the latter earlier than evening types (05:51 p.m. ± 1:24) (F_2,110_ = 9.64—p = 0.0001). The interaction between the two factors was not significant. No significant effects were observed on the MESOR.

**Figure 2:**
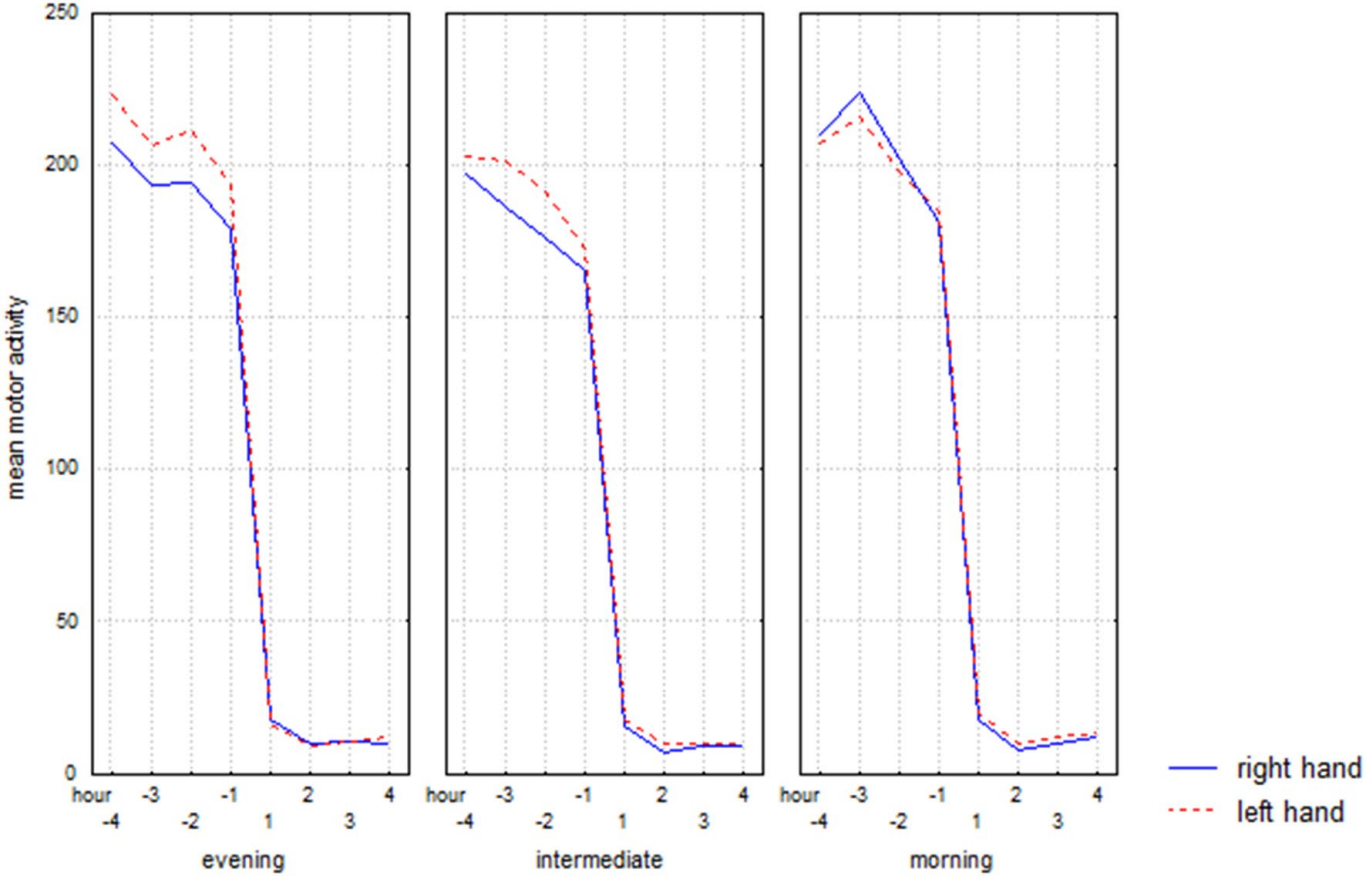
Hourly mean motor activity, over the time interval from four hours prior to bedtime (− 4, − 3, − 2, − 1) to four hours after bedtime (+ 1, + 2, + 3, + 4), of right and left hand in evening, intermediate, and morning types.

As regards the motor activity pattern during the wake-sleep transition (sleep onset), results are summarized in Table 1b and shown in Fig. 2. Overall, the results confirmed a higher mean motor activity level in the left hand in comparison to the right hand before sleep. Performing the Tuckey post-hoc test, such a difference is significant only in evening (− 4 h, p = 0.005; − 2 h, p = 0.002; − 1 h, p = 0.01) and intermediate types (− 3 h, p = 0.00006; − 2 h, p = 0.0001).

**Figure 3:**
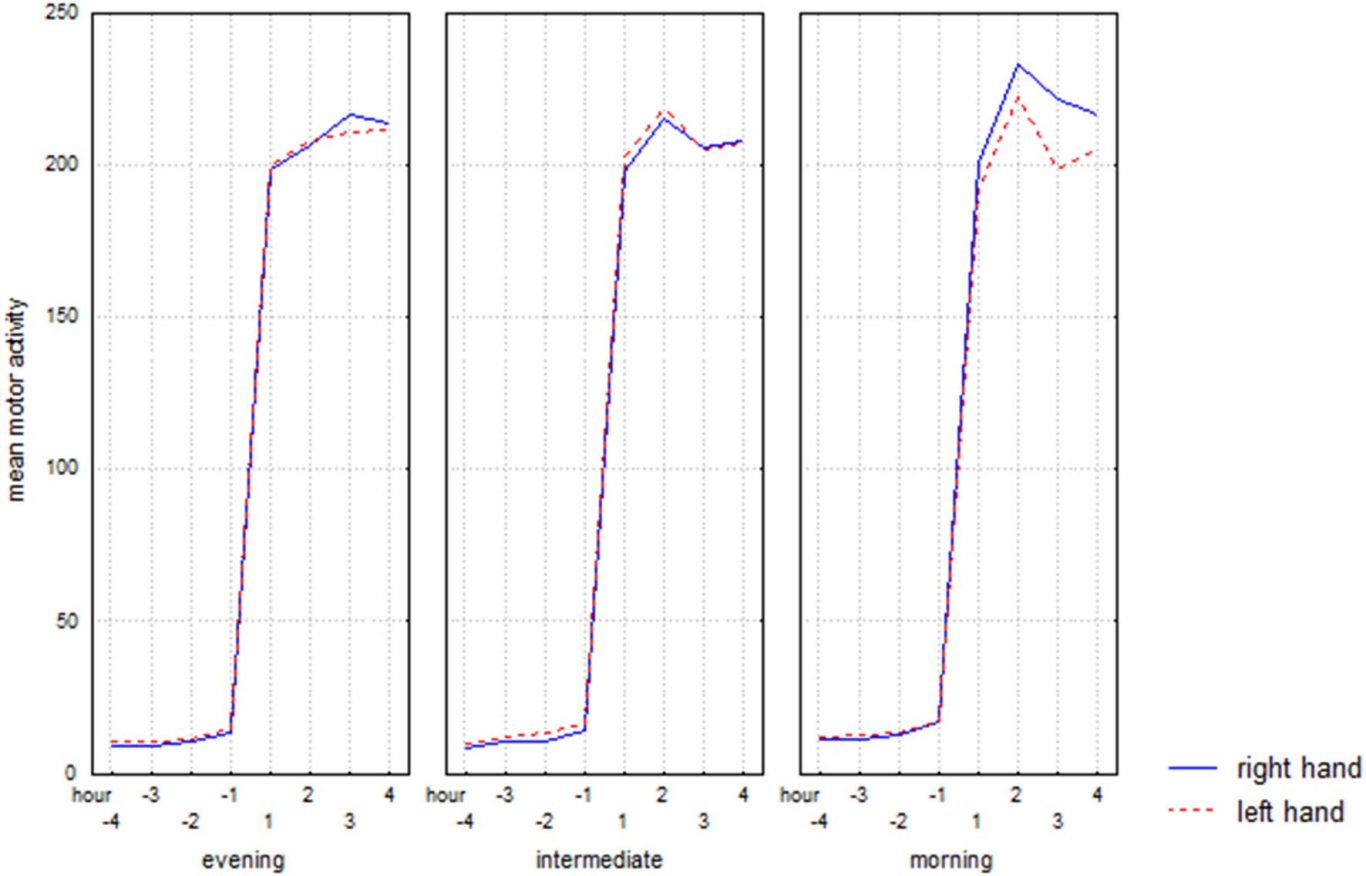
Hourly mean motor activity, over the time interval defined by the four hours before (− 4, − 3, − 2, − 1) and four hours after (+ 1, + 2, + 3, + 4) the get-up time, of the right and left hand in evening, intermediate, and morning types.

Results about the motor activity pattern during the sleep–wake transition (awakening) are summarized in Table 1c and shown in Fig. 3. Post hoc analyses (Tuckey test) only showed a significant difference in morning types with higher mean motor activity in the right hand in comparison to the left (+ 3 h, p = 0.00005).

## Discussion

The results of actigraphic sleep quality and quantity, and the influence of chronotype on sleep phase agree with the literature^22^.

As regards mean motor activity, we confirmed a slight but significant shift (ranging between 15 and 20 min) in the acrophase between the dominant and non-dominant hand late in the evening regardless of chronotype. Looking at mean motor activity over the 24 h, such a phenomenon reaches statistical significance only in evening and intermediate types. It is as if sleep, which occurs much earlier in morning types than the other two circadian types, masks the phenomenon. This interpretation is strengthened by the observation that when we synchronize mean motor activity with wake-sleep transition (sleep onset) such a difference between circadian typologies becomes even more evident. When we synchronize mean motor activity with sleep–wake transition (wake-up) such a difference between circadian typologies becomes specular, i.e., we can see a clearly higher mean motor activity in the dominant hand in morning types but not in evening types.

In conclusion, our results seem to indicate that the relative superiority of the non-dominant hand movements late in the evening could derive from a different circadian phase relationship between the two hemispheres, and that sleep differently masks such a phenomenon depending on the sleep phase (chronotype). Within the theoretical framework of the two-process model of sleep regulation, we could speculate that the left hemisphere is more sensitive to the S process, and for this reason “turns off” before the right hemisphere. On the other hand, the right hemisphere could be more sensitive to C processes and continues its activity late in the evening unaffected by the sleep debt accumulated during the day. Furthermore, we could hypothesize that the left hemisphere is in charge of governing S processes, while the right hemisphere is designated to drive the C process. If this is so, we could conclude that chronotype derives from a different hemispheric balance. Morning types could be more sensitive to the left hemisphere (i.e., S process) and for this reason go to bed early. Evening types could be more sensitive to the right hemisphere (i.e., C process) and for this reason do not go to bed when sleep debt reaches a high level. Such a conclusion partially agrees with previous research that documented morning-types as having more SWA during the first cycle^21^ and a steeper slope of Slow Waves when homeostatic sleep pressure is high^31^ in comparison to evening types. Moreover, such a conclusion also agrees with the observation that morning types show a more marked left-thinking style while evening types are more right-thinkers^32^.

Our conclusions are prevalently speculative and further studies are needed to understand the origin and significance of circadian motor activity asymmetry. The idea that chronotype may arise from a different combination of C and S influences is not new^33–35^. According to Monk^33^, the interaction between the two processes contributes to the generation of the wake-sleep behavior pattern based on the needs of the circadian system and environmental demands. The greater role of process C in evening types is compatible with their higher adaptability to phase change of sleep in comparison to morning-types, while a lesser role of process C in morning types might explain why they are more affected by changes in the sleep–wake cycle^22^.

The strength of this study is certainly its ecology: we studied the participants while they continued to carry out their daily activities outside of the laboratory. At the same time, this is also its principal limitation because the ecological approach should always suggest caution in separating the role of S and C processes^20^. Furthermore, among the limitations the lack of the assessment of potential gender differences, due to the relatively small size of the sample, can be quoted. Such limitation could be overcome by future studies on samples larger in size.

## Data Availability

The data underlying this article cannot be shared publicly for the privacy of individuals that participated in the study. The data will be shared on reasonable request to the corresponding author.
